# The Role of Diet and Nutritional Interventions for the Infant Gut Microbiome

**DOI:** 10.3390/nu16030400

**Published:** 2024-01-30

**Authors:** Giulia Catassi, Marina Aloi, Valentina Giorgio, Antonio Gasbarrini, Giovanni Cammarota, Gianluca Ianiro

**Affiliations:** 1 Department of Translational Medicine and Surgery, Università Cattolica del Sacro Cuore, 00168 Rome, Italy; giulia.catassi@gmail.com (G.C.); antonio.gasbarrini@policlinicogemelli.it (A.G.); giovanni.cammarota@policlinicogemelli.it (G.C.); 2 Pediatric Gastroenterology and Liver Unit, Sapienza University of Rome, Umberto I Hospital, 00161 Rome, Italy; marina.aloi@uniroma1.it; 3 Department of Woman and Child Health and Public Health, UOC Pediatria, Fondazione Policlinico Universitario Agostino Gemelli IRCCS, 00168 Rome, Italy; valentina.giorgio@policlinicogemelli.it; 4 Department of Medical and Surgical Sciences, UOC Gastroenterologia, Fondazione Policlinico Universitario Agostino Gemelli IRCCS, 00168 Rome, Italy; 5 Department of Medical and Surgical Sciences, UOC CEMAD Centro Malattie dell’Apparato Digerente, Medicina Interna e Gastroenterologia, Fondazione Policlinico Universitario Agostino Gemelli IRCCS, 00168 Rome, Italy

**Keywords:** microbiome, pediatrics, diet

## Abstract

The infant gut microbiome plays a key role in the healthy development of the human organism and appears to be influenced by dietary practices through multiple pathways. First, maternal diet during pregnancy and infant nutrition significantly influence the infant gut microbiota. Moreover, breastfeeding fosters the proliferation of beneficial bacteria, while formula feeding increases microbial diversity. The timing of introducing solid foods also influences gut microbiota composition. In preterm infants the gut microbiota development is influenced by multiple factors, including the time since birth and the intake of breast milk, and interventions such as probiotics and prebiotics supplementation show promising results in reducing morbidity and mortality in this population. These findings underscore the need for future research to understand the long-term health impacts of these interventions and for further strategies to enrich the gut microbiome of formula-fed and preterm infants.

## 1. Introduction

The infant gut microbiome is the collection of microorganisms residing in the gastrointestinal tract of newborns and infants (i.e., children between 1 and 23 months of age) [1]. This microbial community is composed of different microorganisms, including bacteria, viruses, fungi, parasites, archaea, and other microbes. Bacteria are the most abundant and diverse group within the gut microbiome [2,3].

Generally, the human gut microbiome is fundamental in shaping the health and well-being of individuals throughout their lifespan [4]. The critical role of this microbial community is even more pronounced during infancy, the period that lays the groundwork for an individual’s long-term health trajectory [5,6]. Multiple recent research works have underscored the significance of the gut microbiome in infants concerning different facets of health and illness [7,8], shedding light on the intricate interactions among microbial populations, nutrition, genetics, and the body’s immune mechanisms [5,6].

The quantitative and qualitative composition of the gut microbiome is heavily dependent on the diet. This is particularly true during the first two years of life due to the many changes that take place during this period of life, including breast and/or formula feeding, weaning and gradual introduction of different solid foods [9,10,11].

This narrative review aims to thoroughly assess the existing scientific literature regarding the impact of diet and nutritional interventions on the infant gut microbiome, exploring the impact of dietary factors on the composition, diversity, and functionality of the gut microbial community in early life. We aim to investigate how specific nutrients, dietary patterns, feeding practices and nutritional intervention (prebiotics, probiotics, and dietary supplements) can influence the establishment and development of a healthy gut microbiome in infants, both in healthy term-newborns and in infants requiring special care (e.g., preterm, very low birth weight, etc.). Finally, we will identify current research gaps and highlight potential areas for future investigation.

## 2. Methods

**Figure 1:**
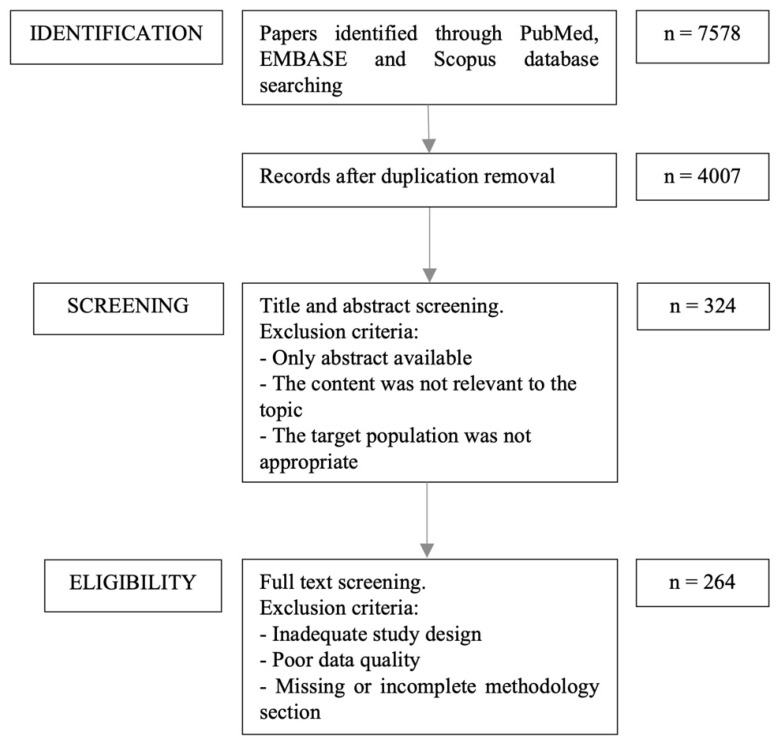
Flow diagram of article selection process.

The authors independently identified the most pertinent published papers that examined the effect of diet on infant gut microbiota. These included observational, retrospective, and prospective studies, as well as case-control, cohort studies, systematic reviews, and meta-analyses. The search was limited to English-language studies published during the last 20 years (2003–2023) and was conducted on PubMed, EMBASE, and Scopus, using the following MeSH-terms: “infant” and “diet” or “food” or “nutrition” and “gastrointestinal microbiome”. The search included all articles that provided sufficient information on the relationship between infant gut microbiota, nutritional interventions, and diet. For every search conducted, the titles of the papers were examined to determine their relevance to the subject. The abstracts of these pertinent papers were then acquired and reviewed, leading to the selection of a subset for a comprehensive review of the entire manuscript. The flow diagram of the article selection process is shown in Figure 1.

## 3. Development of Infant Gut Microbiome and Early Dietary Changes

At birth, the gut of the newborn is considered to be relatively sterile, but it quickly becomes colonized by microorganisms acquired from the environment [12,13]. Studies challenge the sterility dogma of the fetus in utero, revealing bacterial presence in meconium, amniotic fluid, and the placenta [14,15,16], implying a process of maternal-to-offspring microbial transfer in utero [16]. Nevertheless, the crucial colonization phase occurs soon after birth [17].

The consolidation of a steady gut microbiota in humans is typically associated with two major transitions during infancy [18]: the initial transition takes place shortly after birth, during the lactation phase, characterized by the predominance of *Bifidobacterium*. During this phase, the beta diversity is initially pronounced, and the gut microbiota exhibits instability and decreased resistance to alterations compared to the gut microbiota observed in adults [12,18,19]. The subsequent shift ensues during the weaning stage when solid foods are introduced alongside the continuation of milk consumption. This second transition leads to the emergence of an intricate, adult-like microbiome, predominantly populated by the bacterial phyla *Bacteroidetes* and *Firmicutes* [12,19,20]. Following this period, the infant gut microbiota evolves into a more complex, diverse and stable ecosystem [21].

However, prior to reaching this stabilized state, the gut microbiota is more vulnerable to modifications induced by external variables [1,18,20,22]. Elements that contribute to the development of an infant’s gut microbiota include mode of delivery (vaginal birth vs. cesarean section), feeding method (breastfeeding vs. formula feeding), antibiotic exposure, and other environmental factors [8,23,24]. Early-life dietary habits in infants are recognized as significant factors influencing the initial formation of gut microbiota [25,26,27]. This early-life microbial inoculation is of pivotal importance, as these initial colonizers lay the groundwork for future microbial interactions [7,23].

Optimal infant nutrition is crucial for promoting growth and health [28,29]. The World Health Organization (WHO) and the United Nations International Children’s Emergency Fund (UNICEF) have provided specific guidelines for achieving this nutritional goal. They suggest that breastfeeding should commence within the first hour after birth and continue exclusively for the initial six months [30,31]. Beyond this period, breastfeeding should be sustained until the infant reaches two years of age or even longer. Moreover, it is recommended that a complementary diet be incorporated into the infant’s feeding regime no later than when they are six months old [30,31].

## 4. Diet and Nutritional Interventions during Pregnancy

### 4.1. Maternal Diet during Pregnancy 

**Figure 2:**
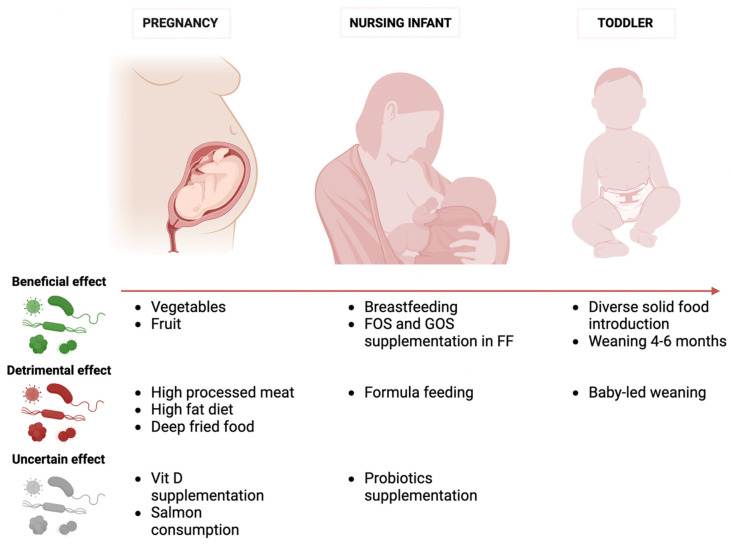
Effects of different microbiome modulators at different stages of the infant life. *FOS*, *fructooligosaccharides*; *GOS*, *galactooligosaccharides*; *FF*, *formula feeding*.

Scientists are increasingly intrigued by the impact of dietary and nutritional interventions during pregnancy on the development of the infant gut microbiome. Figure 2 summarizes the effects of different microbiome modulators at different stages of the infant’s life. According to the Developmental Origins of Health and Disease Hypothesis (DOHaD), neonatal gastrointestinal colonization during the first 1000 days after birth is an essential stage in growth and development, strongly impacted by maternal diet [32]. Numerous research projects have examined the relationship between a mother’s diet and her baby’s intestinal microbiota using food frequency questionnaires, yielding inconsistent findings [33,34]. The dietary elements analyzed in mothers included general eating habits as well as particular nutritional elements [32,35,36,37]. After adjusting for variables like demographics, delivery mode, and breastfeeding status, no significant independent impact on the infant microbiome was found [33]. Urwin et al. [38] discovered that a mother’s bi-weekly intake of salmon did not notably affect the gut microbiota of both the mother and her child. In this context, a study by Garcia Mantrana et al. [39] explored the specific relationship between maternal diet during pregnancy and the composition of both maternal and neonatal gut microbiota, employing 16S rRNA gene sequencing. This study revealed that dietary components such as fiber, lipids, and proteins were linked to specific clusters of gut microbiota in both mothers and their children. A considerable body of evidence comes from studies investigating the role of vitamin D during pregnancy. Using data from the same group of the Vitamin D Antenatal Asthma Reduction Trial (VDAART), it was observed that prenatal dietary habits classified as “healthy”—marked by abundant vegetable consumption and minimal intake of processed meats and deep-fried foods—were associated with a higher alpha diversity in the gut microbiota of newborns. Yet, these variations lost statistical significance upon demographic adjustments and consideration of the infant’s feeding mode [40,41]. In a distinct study, the VDAART team discovered no relation between supplementation of vitamin D during pregnancy and the diversity of gut microbes in infants aged between three to six months [42]. The KOALA birth cohort research also reported negligible connections between mothers’ vitamin D supplementation and 25-hydroxyvitamin D concentration and their infants’ intestinal microbiota. The only exception noted was an increase in *Bifidobacteria* counts in the stools of one-month-old babies following maternal vitamin D supplementation. No further significant links were evident in the models after adjustments [43].

Additionally, a high-fat diet appears to play a role in the infant microbiome. Chu and colleagues observed that regardless of the mother’s weight, babies born to mothers with a high-fat dietary intake exhibited a different meconium composition and a lower proportion of Bacteroides a few weeks after birth in comparison to those in the control group [44].

A key component of a mother’s diet that seems to have a notable impact on her baby’s intestinal microbiota is the consumption of fruits and vegetables [45]. Lundgren et al. [46] noted a substantial link between a mother’s fruit intake and the makeup of her baby’s fecal microbiome at six weeks, but this connection was only evident in babies born through vaginal delivery and who were solely breastfed. In line with this, a pilot study by Fan et al. [47] found a significant correlation between the level of maternal fruit and vegetable consumption and the infant gut microbiome composition at two months of age. It is indeed important to recognize that the gut microbiome’s structure is affected by the consumption of dietary fibers, which certain bacteria metabolize to produce short-chain fatty acids (SCFAs), including acetate, propionate, and butyrate [48,49,50]. In line with this, this study showed a pronounced increase in *Cutibacterium*, *Parabacteroides*, and *Lactococcus* in the intestinal microflora of infants exposed to a diet rich in fruits and vegetables during gestation. Conversely, an increased occurrence of inflammatory strains, like *Sutterella*, was associated with a lower intake of these foods [47].

### 4.2. Probiotics and Prebiotics during Pregnancy and Lactation

The scientific community has shown growing interest in the potential benefits of altering the infant gut microbiome by adding probiotics or prebiotics during pregnancy and breastfeeding. An extensive review of 17 interventional trials investigated the effects of administering probiotics to mothers during pregnancy on the newborn’s intestinal microbiota [32]. Supplementation was carried out using probiotics containing at least one species of *Lactobacillus* and/or *Bifidobacterium*, delivered in various forms, such as capsules, powder, milk, yogurt, or oil droplets. The supplementation was given to women during their second or third trimester of pregnancy. This review showed that the administration of probiotics to pregnant and lactating mothers has a significant impact on establishing the newborn’s gut ecosystem and influencing the microbial composition of maternal breast milk. However, there is scarce evidence that this intervention determines sustained colonization or can significantly influence the overall diversity of the infant microbiome [51,52,53]. While some studies report a higher abundance of supplemented species at the time of intervention [52,53,54,55,56,57], other research indicated no notable changes in the abundance of species added through supplementation following the discontinuation of the probiotic treatment [58,59,60,61]. A recent metanalysis of seven studies affirms that supplementing mothers with probiotics during pregnancy and breastfeeding leads to the infant’s gut being colonized by the species provided [51]. The abundance of these species reached its zenith during the first month of life, prior to a consistent decline, potentially attributed to the competitive interplay with other emergent bacterial populations. Overall, in a couple of randomized studies evaluating changes in ecological metrics after treatment with probiotics, no notable statistical variances were observed in alpha and beta diversity at various intervals when comparing probiotic supplementation with placebo [53,62].

Only a few (but well-designed) studies evaluated the effect of prebiotics, mainly galactooligosaccharides (GOS) and long-chain fructooligosaccharides (lcFOS), on infant gut microbiota [51,63]. A placebo-controlled RCT of 52 pregnant women explored the possible effects of GOS supplementation on maternal intestinal microbiota, inflammation and energy pathway [64]. Individuals receiving GOS had an increased level of *Paraprevotella* and *Dorea* but less *Lachnospiraceae* compared to those in the placebo group [64]. Another RCT found a positive correlation between maternal FOS intake and bifidobacterial count in the mothers but not in the neonates [65]. By contrast, a further RCT found no differences in *Bifidobacteria* and *Lactobacilli* abundances, alpha and beta diversity between mothers receiving GOS/lcFOS and those receiving placebo [66].

In conclusion, there is currently insufficient evidence to support the practice of maternal supplementation with probiotics or prebiotics to modulate the infant gut microbiome, also considering that these interventions appear to have only a temporary colonization effect that does not remain following the termination of treatment [32,67].

## 5. Diet and Nutritional Interventions in Early Life

### 5.1. Milk Feeding in Early Life

The maturation of the gut microbiota in early life is deeply influenced by the methods of infant nutrition, which are breast milk and formula feeding [68]. The type of feeding significantly influences the composition and function of the infant gut microbiota mainly because of differences in nutrient composition, particularly related to the Human Milk Oligosaccharides (HMOs) [69].

### 5.2. Role of Breastfeeding in Shaping the Infant Gut Microbiome

The primary role of breast feeding in establishing a healthy infant gut microbiome has been increasingly recognized in recent years [5]. Breast milk contains different components, including proteins, fats, carbohydrates, and immunoglobulins [70]. A significant component of breast milk is the HMOs, such as GOS, which undergo only partial digestion in the small intestine, mainly reaching the colon [71]. In the colon, HMOs are fermented, largely by *Bifidobacteria*, resulting in the production of SCFAs [72] that inhibit the growth of opportunistic pathogens, specifically belonging to the Clostridiaceae, Enterobacteriaceae, and Staphylococcaceae families [25,73,74]. Sakurama and colleagues showed that *Bifidobacteria* produce an enzyme, lacto-N-biosidase, that contributes to the digestion of GOS [75]. As shown by Matsuki et al. [76], Bifidobacterium numbers increase, HMO content in stool decreases, and the levels of acetic and lactic acid increase in one-month-old infants. Consequently, HMOs exhibit a pronounced prebiotic impact by selectively fostering the growth of a *Bifidobacterium*-dense microbiota.

*Bifidobacteria*, particularly the *Bifidobacterium infantis*, exhibit a direct correlation with the levels of mucosal Immunoglobulin A (IgA) secreted by the gut [77]. Additionally, this bacterium is known for its anti-inflammatory properties.

Therefore, the synergy between HMOs and *Bifidobacteria* not only enhances the variety and equilibrium of the baby’s intestinal microbiota but is also crucial in supporting the host’s immune system and general well-being. Moreover, remnants of HMO metabolism, such as fucose, lactate, and 1,2 propanediol, as well as aromatic amino acid-derived co-HMO metabolism products like indolelactate and 4-hydroxypheyllactate, are typically present in breastfed (BF) infants [1,78].

Additionally, human milk is also a source of bacteria that colonize the infant gut [79]. Mother-to-infant transmission studies, accounting for both cultured and non-cultured bacteria, provide strong evidence that this bacterial transfer takes place through breastfeeding [24,80]. This transmission has been verified by detecting the same bacterial strains in both maternal milk and the stool of breastfed infants [81]. Furthermore, research by Pannaraj and colleagues [82] suggests that bacterial transmission via breast milk has a more profound influence on the early bacterial colonization of a newborn than the bacteria from the areolar skin.

Human breast milk is comprised of a diverse array of microbiota, encompassing both skin-related and non-skin-related Gram-positive bacterial strains [83]. Notably, Streptococci (specifically *S. mitis* and *S. salivarius*) and coagulase-negative Staphylococci prevail in both human milk and stool of breastfed babies [84]. These microorganisms can compete with undesirable pathobionts, such as *Staphylococcus aureus*, for space and resources within the infant gut.

The origin of the microbial population in breast milk remains uncertain. The entero-mammary pathway theory suggests that immune cells selectively transport bacteria from the gut to the mammary gland [85]. This idea is supported by data indicating a resemblance in the bacterial profiles of a mother’s feces and her breast milk [86]. This hypothesis is further supported by clinical studies finding probiotic strains previously ingested by the mother in her breast milk [87,88].

Infant feeding also influences significantly the host gene expression, as demonstrated by transcriptomic studies conducted on intestinal epithelial cells [89]. It has been observed that breastfeeding increases the transcription of genes related to immunological processes and metabolic functions [89]. Breastfeeding plays a key role in rectifying disruptions in the infant’s gut microbiota resulting from cesarean birth, highlighting its essential function in forming a robust intestinal microbiota, regardless of the method of delivery [90].

### 5.3. Impact of Breastfeeding Duration and Exclusivity

The duration and exclusivity of breastfeeding are major drivers of infant gut microbiota composition [91]. Both exclusive breastfeeding (EBF), defined as the consumption of only breast milk without any additional formula milk, food, or drink, not even water, and its duration, shape specifically the infant gut microbiota [91,92,93,94]. A meta-analysis of seven studies revealed that during the first 6 months of life non-exclusively breastfed infants exhibited consistently higher gut bacterial diversity and microbiota age compared to exclusively breastfed infants [92]. Furthermore, relative abundances of Bacteroidetes and Firmicutes and their respective energy pathway were consistently higher in non-exclusively breastfed infants [92]. These differences persisted until 2 years of age. In the CHILD study [91], the relationship between exclusive breastfeeding and duration of EBF and the prevalence and relative abundance of different bacteria in the infant gut, represented by amplicon sequencing variants (ASVs), was analyzed, with notable differences in the overall relative abundance of ASVs at 3 and 12 months in exclusive vs. non-exclusive BF. In a recent work by Chichlowski [94], the gut microbiome of EBF infants was less diverse but more stable compared to formula-fed infants. *Bifidobacterium*, known for selectively using HMOs as growth substrates, was the dominant genus in the infants’ stools at all points in time, regardless of EBF duration. Infants who experienced EBF for more than six months exhibited a greater relative abundance of *Bifidobacterium bifidum* compared to those who were EBF for less than three months [94].

Laursen identified a positive correlation between the duration of breastfeeding and the occurrence of *Bifidobacterium*, *Veillonella*, *Megasphaera*, *Haemophilus*, lactic acid bacteria, and Enterobacteriaceae. Conversely, longer breastfeeding duration had a negative effect on the abundance of *Lachnospiraceae* and *Ruminococcaceae*, bacteria known for breaking down complex carbohydrates [95].

### 5.4. Role of Formula Feeding in Shaping the Infant Gut Microbiome

Formula-fed (FF) infants show more diverse colonization compared to their breastfed counterparts [96]. Infants who are FF show a greater prevalence of *Clostridiales* and *Proteobacteria* in their gut microbiome [79]. Additionally, the gut microbiota of these infants tends to have a higher concentration of *Atopobium* and *Bacteroides* but less *Bifidobacteria* compared to breastfed infants [97]. Formula feeding has also been observed to decrease the overall quantity of gut bacteria while simultaneously increasing the diversity within the gut microbiome [94,98].

This difference in microbiota composition is primarily attributed to the absence of HMOs and the increased protein content in formula milk. Infant formulas often contain supplemental FOS and/or GOS, but these are not as selective as HMOs [99]. They can stimulate the growth of various bacterial species, leading to a significantly different microbiota composition compared to that seen in breastfed infants [100,101].

Interestingly, the gut microbiota of FF infants, even when the formula contains GOS, show a predominance of proteolytic over saccharolytic metabolism [102,103]. This is evidenced by the elevated concentrations of protein breakdown byproducts [97]. Unfortunately, some of these metabolites can be converted in the liver into detrimental metabolites, such as p-cresol-sulfate and phenylacetateglutamine; these compounds can contribute to enterocyte toxicity, promote inflammation and increased gut permeability and disrupt normal metabolic functions by competing with other substances for sulfation in the liver, a pathway used to detoxify a variety of compounds [104,105].

### 5.5. How Changes in the Composition of Infant Formula Can Modulate Infant Gut Microbiota

Efforts to promote the development of a gut microbiome in FF infants that closely resembles that of a breastfed infant in order to emulate health advantages conferred by breast milk include the supplementation of infant formula with prebiotics, probiotics or symbiotics [106,107,108], which are synergistic combinations of both.

### 5.6. Prebiotics

Numerous research efforts have been conducted to explore the impact of prebiotic addition on the composition of the infant gut microbiome [99,109]. Research has demonstrated the advantages of enriching infant formula with HMOs like 2′ fucosyllactose and lacto-N-neotetraose [110]. The goal of this strategy is to replicate the positive impacts that breast milk has on the intestinal microbiota. Initial studies have shown promising results, as the gut microbiota of infants fed with HMO-supplemented formula showed a greater resemblance to that of breastfed infants [111]. These supplements not only support optimal growth in infants, but also promote the growth of beneficial *Bifidobacteria*, achieving a gut microbial composition closer to that of breastfed infants. The supplementation of infant formula with GOS and FOS can lead to an increased abundance of *Bifidobacteria* and lower fecal pH, mirroring attributes of breastfed infants [112].

Although infant formula products are engineered to replicate the macronutrient profile of human milk, currently, the majority of them do not incorporate substantial levels of prebiotics and/or probiotics, as reported by Salminen et al. in 2020 [113]. Babies fed with formula enhanced with HMOs exhibited increased *Bifidobacteria* and decreased *Enterobacteriaceae* and *Peptostreptococcaceae* [107]. A study by Borewicz [99] compared the fecal microbiota composition in infants who were breastfed with that of babies fed with an infant formula fortified with prebiotics (GOS and/or FOS) or receiving mixed feeding. These findings were compared with those from infants who were given conventional formulas. By next-generation sequencing analysis, this study demonstrated a bifidogenic effect of prebiotic-fortified formulas as compared to traditional formulas. Infants who were fed formulas fortified with prebiotics showed gut microbiota compositions that were more similar to those found in breastfed babies. This was not the case in formula-fed infants who were given formulas without any added prebiotics. This study also demonstrated lower bifidogenic activity in formulas combined with breastmilk feeding, suggesting a possible interference between the components of the two [99].

The addiction of bovine milk-derived oligosaccharides (MOS) to infant formula was evaluated in a three-arm RCT including a control group fed on regular cow milk-based formula, an experimental group receiving the same formula but with added MOS, and a reference group of exclusively breast milk-fed infants [114]. The overall gut microbiota composition in the experimental group showed more similarities with that of breast milk-fed infants than with the control group. *Bifidobacteria* were found in higher abundance in the experimental group compared to the control group. Moreover, infants born via cesarean in the experimental group also showed a microbiota composition that was more similar to breast milk-fed and vaginally born infants than to the control group infants. By the age of 4 months, counts of harmful bacteria, *Clostridioides difficile* and *Clostridium perfringens*, were significantly reduced in the experimental group than in the control group. The experimental group also showed twice the amount of fecal secretory IgA compared to the control group.

Two comprehensive systematic reviews carried out by Rao et al. [109] and Mugambi [115] examined the effects of adding prebiotics to formula milk. Both showed higher stool colony counts of *Bifidobacteria*, regardless of differences in dosage, duration of supplementation, and method of reporting results. However, three specific studies using supplementation with GOS, FOS or a GOS/FOS mix found no difference in *Bifidobacteria* levels between the infant formula-supplemented groups and their controls [116,117,118]. Prebiotic supplementation had an inconsistent impact on [117,118,119,120] while decreasing the levels of *C. difficile* [121,122]. A double-blinded RCT comparing an infant formula supplemented with a symbiotic composed of bovine MOS and the probiotic *Bifidobacterium animalis* vs. the same formula alone caused a significant increase in *Bifidobacteria* abundance and lower microbiota diversity in the experimental group vs. controls, similarly to breastfed infant [123].

### 5.7. Probiotics

Currently, the most frequently examined and utilized probiotic species belong to the *Lactobacillus*, *Bifidobacterium*, and *Saccharomyces* genera [124,125]. Researchers are also exploring the potential application of bacteria extracted from breast milk to develop infant formulas that closely resemble the nutritional composition of natural breast milk [5]. Supplementation of the formula with *Bifidobacterium* species and/or lactic acid bacteria, such as *Lactobacillus* strains, is deemed secure and generally accepted [126,127,128] and may potentially enhance their immune response [129,130].

Studies investigating the effect of probiotic supplementation of infant formulas did not find a strong correlation between fecal *Bifidobacterium* concentration and *Bifidobacterium* supplementation [131,132,133]. *Bifidobacteria* colonization in the infant gut was indeed found to be unstable over time, most likely due to competition among members of the gut microbial [134,135]. This finding has been supported by a systematic review [120] of 12 RCTs, reporting that supplementation of probiotics did not increase the counts of *Bifidobacteria* or *Lactobacilli* nor decreased the levels of pathogens such as *Bacteroides* and *E. coli*. [115].

In a recent observational study, neonates undergoing varied probiotic administration for six months showed an elevation in stool *Bifidobacteria* levels only during the first week after birth, implying that probiotics might potentially expedite the initial colonization of this taxon, together with a concomitant reduction in the Enterobacteriaceae family, without differences in alpha diversity [136]. Regardless of the probiotic species, fecal *Lactobacillus* levels were higher in infants supplemented with a probiotic [136,137]. Another investigation revealed that healthy infants given formula supplemented with *Lactobacillus rhamnosus GG* (LGG) showed a greater frequency of *Lactobacilli* colonization compared to those who were fed with a standard formula [138]. Additionally, in very low birth weight infants, the supplementation of *Bifidobacterium breve* Bb12 favored gut colonization by the added bacteria and expedited the growth of *Lactobacilli* compared to those infants who did not receive the probiotic supplement [139].

To foster a beneficial gut microbiota, the most opportune time for administering probiotics is prior to the establishment and colonization of individual microbial taxa [140]. This crucial window is typically within the initial months of life. Nonetheless, the colonization timings vary across different microbial taxa [141]. Therefore, identifying these specific periods of opportunity for each taxon is of paramount importance. However, the optimal duration of probiotic supplementation required to guarantee a protracted beneficial impact on gut microbiota remains unclear.

### 5.8. Introduction of Complementary Foods

During the fourth month of life, the infant’s renal and gastrointestinal systems reach physiological maturation, enabling them to process non-milk alimentary substances [142]. Upon reaching the sixth month, the nutritional and energetic benefits procured solely from breast milk become insufficient to meet the growing metabolic demands of the infant [143]. Thus, the inclusion of complementary food is needed for the appropriate somatic and neurodevelopmental trajectory [142,144].

The implementation of complementary feeding presents a heterogeneous pattern across Europe and worldwide. Certain European regions, exemplified by the UK and Sweden, adhere to the World Health Organization’s endorsement of starting such feeding regimens from six months. However, other territories, including Belgium and Spain, advocate for the initiation of these diets between the fourth and sixth month, a strategy that is in alignment with the guidelines of the European Society for Paediatric Gastroenterology, Hepatology, and Nutrition (ESPGHAN) Committee on Nutrition [145]. According to this committee, the introduction of complementary alimentary substances should not precede the fourth month and should not be delayed beyond the sixth month, including those containing potential allergenic substances.

### 5.9. Diversity of Solid Food Introduction

Despite remarkable advancements in our knowledge of early-life gut microbial interaction and our growing understanding of the microbial capacity to metabolize various dietary compounds, the understanding of the effects of diet on gut microbiota during the complementary feeding period is still limited [146].

The shift from exclusive milk feeding to the inclusion of family foods in the infant’s diet corresponds with substantial changes in the gut microbiota [147]. During this period, the alpha diversity increases, with a shift from *Bifidobacterium*-dominant community to *Bacteroidetes*- and *Firmicutes*-dominant communities [148]. For instance, there is a rapid decrease in the population of *Bifidobacterium* species that can degrade HMO [20,149]. Simultaneously, there is a significant increase in diversity and the emergence of *Bacteroidaceae*, *Lachnospiraceae*, and *Ruminococcaceae* species, reflecting the more complex diet that comes with the introduction of fibers and new proteins [150,151]. A longitudinal study by Stewart et al. [152] reported a clear increase in gut microbial diversity after the introduction of solid foods. Further, this increased diversity correlated with enhanced immunological and metabolic development in infants, suggesting the potential health benefits of diverse solid food introduction. A recent study by Pannaraj et al. [82] provided similar findings, reporting the association of diversified solid food intake with the enrichment of specific microbial groups, particularly *Bifidobacterium* and *Bacteroides*.

Diverse solid foods act as new sources of microbiota-accessible carbohydrates, therefore stimulating the growth of beneficial taxa such as *Bifidobacterium*, *Lactobacillus*, and *Bacteroides* [153]. These microbes produce SCFAs, such as butyrate, propionate, and acetate, promoting a healthy gut environment and influencing the immune system [154]. The introduction of fruits and vegetables, rich in fermentable fibers, leads to an increase in beneficial microbes like *Bacteroides* and *Bifidobacterium* [155]. On the other hand, protein-rich foods like meats and eggs can stimulate proteolytic microbes such as *Clostridium* and *Streptococcus* [156]. Hence, the introduction of a diverse diet could ensure a balance between these microbes, leading to a more resilient and healthier gut microbiota.

Since gut microbes primarily derive their energy from dietary fibers and secondarily from proteins/peptides, these macronutrients are likely to have the greatest influence on the microbial composition [49,157]. The primary outputs of metabolizing dietary fiber include SCFAs like acetate, butyrate, and propionate [158,159]. High levels of acetate are generated during the initial stages of infancy, whereas the levels of butyrate and propionate start at a markedly reduced state, subsequently elevating as the infant grows older [9]. Correspondingly, the products of protein degradation, notably branched-chain fatty acids (BCFAs), remain essentially unobservable during the lactation period yet exhibit a parallel trajectory of augmentation with advancing age [95]. These changes align with the beginning of solid food intake and the end of breastfeeding [160]. In agreement with the typical gut microbiota developmental pattern, key species within the *Lachnospiraceae* and *Ruminococcaceae* families produce butyrate, while *Bacteroides* species are common propionate producers [161]. These species possess a comprehensive array of enzymes for breaking down dietary fibers into these SCFAs [162]. Moreover, certain species more abundant in older infants, such as *Bacteroides* and *Clostridium*, might employ a range of amino acids derived from dietary proteins to produce BCFAs [163]. Thus, complementary feeding might have a causative effect on microbiota composition and metabolism [69,164].

In a study of nine-month-olds infants, the diversity of gut microbiota was found to increase with the introduction of solid food, particularly fibers and protein, independent of whether the infants were breastfed or formula-fed [165]. A study by Marrs [26] suggests that the introduction of allergenic food, in conjunction with continued breastfeeding between 3 to 6 months of age, resulted in the increase of the overall gut microbiota Shannon diversity. Specifically, this diversification was characterized by the emergence of various microbial taxa, notably *Prevotellaceae* and *Escherichia/Shigella*. Of note, the presence of *Prevotella* has been linked with high-fiber diets [150].

Elevated protein intake has been associated with a heightened abundance of *Lachnospiraceae* and a decrease in saccharolytic organisms, such as those in the *Bifidobacteriaceae* family [166]. Simultaneously, the consumption of fiber was linked to an increase in the proportions of Prevotellaceae [167,168].

### 5.10. Timing of Solid Food Introduction

The timing of complementary food introduction is known to influence gut microbiota composition. A study by Bäckhed et al. [169] suggests that the delayed introduction of solid food could cause a lag in microbial maturation and increase susceptibility to allergies and obesity. On the other hand, an earlier introduction could expose infants to potential pathogens and allergens [170,171,172]. Hence, the timing of solid food introduction should balance between these risks and benefits.

Differding and coworkers found that the introduction of complementary feeding before 3 months of age can lead to enhanced microbial diversity and a higher concentration of fecal butyrate and that these effects may continue up to the age of 12 months [148]. In an RCT comparing traditional spoon feeding to a baby-led approach (involving self-feeding with complementary “finger foods”), the authors found that babies weaned through a baby-led approach were introduced to solid foods approximately 20 days beyond the initial six months (at the age of seven months) [173]. At this age, their consumption of both vegetables and fibrous nutrients was markedly reduced.

By contrast, Laursen and colleagues discovered that the length of time infants were breastfed had a greater influence on both the variety and the proportion of intestinal microbiota and their overall microbial richness at the age of nine months than when they began eating solid complements [147]. This conclusion aligns with the latest findings from Bäckhed et al. [169], which indicate an increase in *Lachnospiraceae* populations correlating with increased consumption of household meals, as opposed to a decline in *Bifidobacteriaceae* numbers. This alteration likely mirrors the dietary shift from mother’s milk, which is rich in *Bifidobacteriaceae*, to solid foods typical of late infancy that are abundant in fiber and protein, thus supporting the growth of *Lachnospiraceae* species [147].

In another study, Differding and coworkers [174] investigated how the timing of introducing complementary foods can significantly affect the infant’s gut microbiota composition, in turn potentially impacting their gut health and overall nutrition: *Ruminococcus bromii*, which is able to digest resistant starches [19] was found in greater amounts in infants who were breastfed for less than four months and given complementary foods early. In infants fed with a diet rich in resistant starches, *R. bromii* could potentially outperform other commensal bacteria that are not as efficient in energy extraction, potentially causing a shift in metabolic processes and dysbiosis. Additionally, these infants had a reduced number of *Bifidobacterium animalis*, a dominant bacterial species in young gut ecosystems, which generally diminishes with the infant’s growth and the onset of weaning [152,174]. An increased presence of *Bifidobacterium animalis* may be advantageous for the gastrointestinal health of infants, as indicated by a randomized controlled trial which demonstrated that its supplementation reduced the levels of fecal calprotectin (an indicator of gut inflammation) and decreased gastrointestinal leakiness in infants born before term [175].

## 6. The Effect of Diet and Nutritional Interventions on Gut Microbiota in Preterm Infants

Preterm infants born earlier than 32 weeks of gestation experience an atypical beginning of life. Their conditions often require placement in nearly sterile incubators, greater frequency of cesarean deliveries, and antibiotic use, combined with the inability to breastfeed, necessitating complex nutritional supplementation and parenteral nutrition that circumvents the gastrointestinal tract [176].

While these measures are crucial for maximizing survival in this susceptible population, they invariably result in an abnormal gut microbiome and altered microbial-host interaction, as compared to full-term infants [177,178]. Preterm infants also have an immature gastrointestinal tract that is abruptly exposed to a wide variety of microbes following birth [178]. Early colonization of the gut in these infants differs significantly from that in term infants, often characterized by lower microbial diversity, lower abundance of *Bifidobacteria* and *Bacteroidetes*, higher abundance of *Proteobacteria*, and greater colonization by pathogens [164,179]. Premature babies greatly benefit from maternal breast milk, which significantly reduces the risk of necrotizing enterocolitis (NEC), a common gastrointestinal disorder among preterm infants [180,181]. However, this does not entirely eliminate the risk, indicating that various other factors, including the variability in the composition of human milk, also play a role. Extremely premature newborns are unable to engage in direct breastfeeding, which leads to the necessity for extracting, cooling, and tube-feeding breast milk. This procedure, unfortunately, can result in an accumulation of harmful bacteria while simultaneously diminishing the presence of protective *Bifidobacterium* [182,183,184]. Appropriate nutrition, especially in the early stages, is vital for these infants. Studies have shown that early and higher nutrient provision, particularly through the mother’s own milk, not only supports growth [185] but also positively influences clinical outcomes like neurodevelopment and reduces the risk of complications typical of this condition, such as the bronchopulmonary dysplasia and retinopathy of prematurity [186,187,188]. In this context, a secondary analysis of a randomized controlled trial provides further insight into the impact of early versus delayed initiation of enteral feeding on the gut microbiome of extremely preterm infants [189]. This study found that early enteral feeding was associated with an increased presence of three specific bacterial genera in the gut: *Bilophila*, *Veillonella*, and *Lactococcus*. *Bilophila* species, which can proliferate with dietary changes, have been linked to both beneficial and potentially harmful effects. *Veillonella* spp., known as colonizers of the oral cavity and gastrointestinal tract, are generally considered harmless and potentially beneficial. *Lactococcus*, a lactic acid-producing bacteria, is noted for its potential as a probiotic, particularly in reducing the risk of NEC in preterm infants. However, the study did not find evidence that early human milk diets enhance the diversity of the gut microbiome in these infants, possibly due to minimal differences in feeding volumes between early and delayed feeding groups [189].

A prospective longitudinal study [164] evaluated the gut microbiome patterns in preterm infants during their first 30 days of life in the neonatal intensive care unit, finding that the composition of the gut microbiome varied significantly between individuals, with *Proteobacteria* being the most prevalent phylum. During the first 30 days post-birth, the gut microbiome of these preterm infants demonstrated an initially low diversity index that gradually increased daily. This pattern of microbial community was marked by an increased presence of *Clostridium* and *Bacteroides* and a corresponding decline in *Staphylococcus* and *Haemophilus* over time [164].

Two primary factors influence the gut microbiome development in preterm infants: time (days since birth) and the intake of breastmilk from their own mothers [190].

Alterations or delayed maturation of gut microbiota, primarily characterized by an increase in *Proteobacteria* and *Firmicutes*, have been associated with conditions such as NEC and late-onset sepsis in preterm infants [191].

Infants who are fed their mother’s breast milk have significantly more diverse gut microbiota and a greater abundance of *Clostridiales* and *Lactobacillales* than infants who are fed human donor milk and/or formula [164]. Conversely, those infants fed non-mothers breast milk displayed a distinct microbiome community, predominantly marked by *Enterobacteriales* during their initial 30 days of life [192].

Preterm infants fed by mother’s breast milk do not show a significant increase in *Bifidobacteria* but display a higher abundance of Firmicutes bacteria [193]. This finding could be explained by the fact that the breast milk produced by mothers of preterm infants differs significantly from that of mothers of full-term infants, which might influence the premature gut differently [194]. Moreover, preterm infants may respond to HMOs in a distinct way compared to full-term infants [195].

The same findings are reported in a study conducted by Cong et al. [164]; they discovered that infants fed with breast milk displayed increased populations of *Clostridiales*, *Lactobacillales*, and *Bacillales* while exhibiting decreased numbers of *Enterobacteriaceae*. Conversely, infants who were fed with donor human milk and formula milk showed an elevated proportion of Enterobacteriaceae. The study revealed that infants who were breastfed exhibited notably higher alpha diversity in their gut microbiota. In terms of beta diversity, the method of feeding emerged as the primary factor influencing variation, followed by other variables such as gender, gestational age, postnatal age, antibiotic usage, and premature membrane rupture.

### 6.1. The Role of Diet in Shaping the Gut Microbiota in Preterm Babies

Human milk contains a variety of HMOs that are not found in standard preterm formulas [195]. HMOs have been shown to have critical roles in epithelial function and immune development [196,197]. For instance, disialyllacto-N-tetraose (DSLNT), an HMO, has been associated with protection against NEC [198]. Furthermore, another HMO, 2′-fucosyllactose (2′FL), has been shown to suppress inflammation [105]. Given the crucial role of the gut microbiome in NEC and advancements in sequencing technologies, several studies tried to identify specific bacteria or bacterial combinations associated with NEC onset [199]. However, no consistent bacterial association has been found, although a pattern of higher Proteobacteria abundance and lower *Bifidobacterium* spp. diversity appears to be frequent in infants with NEC [200].

Recent research correlating the HMO composition in human milk with changes in microbiota evolution shows that low concentrations of DSLNT in the milk are associated with slower progression of microbial maturation, that are typically abundant in *Bifidobacterium* spp. [198]. These studies underscore the significance of the diet-microbe-host interaction, though it remains challenging to establish causality from observational studies.

A meta-analysis revealed a reduced occurrence of NEC in preterm babies when donor human milk (DHM) was used as the exclusive feed compared to formula milk [201]. Of the two most important RCTs, one indicated a lower incidence of NEC with the use of DHM, while the other reported no notable difference. In a recent RCT by Embleton and coworkers [202], 126 preterm babies were randomly assigned to pasteurized human milk and human milk-derived fortifiers or bovine formula and bovine-derived fortifier, without significant differences in terms of microbiome composition and alpha diversity and in the incidence of NEC or any other key neonatal health problems.

### 6.2. Effect of Probiotics or Prebiotics to Prevent Morbidity and Mortality in Preterm Infants

Supplementation of probiotics in preterm infants is seen as a potential strategy to assist the initial colonization of the neonatal intestine [203,204]. In addition, probiotic supplementation was reported to have numerous beneficial and anti-inflammatory effects [205,206].

According to a recent systematic review of 67 RCTs and a recent metanalysis of 70 studies, supplementation with probiotics, specifically those containing *B. infantis*, significantly reduced the risk of NEC, Late-Onset Sepsis (LOS), and all-cause mortality in preterm infants [207,208]. This aligns with the results of another systematic review by Beghetti and coworkers, who observed that a strain of probiotics, *B. lactis* Bb-12/B94, was associated with reduced risk of NEC in both exclusively human-milk-fed and non-exclusively milk-fed; more favorable results were seen in infants fed exclusively with human milk [203]. However, some concerns and challenges have been identified. There have been rare reports of fungal infections caused by probiotic contamination, indicating a need for stringent pharmaceutical standards for probiotic production and maintenance [205,209].

A systematic review and network meta-analysis [210] highlighted that both single- and multiple-strain probiotics exhibit superior effectiveness compared to a placebo in mitigating mortality and morbidity rates in premature infants, especially a blend of at least one *Lactobacillus* species and one *Bifidobacterium* species is the optimal strategy for preventing all-cause mortality and stage II NEC [210].

A meta-analysis conducted by Chi et al. compared the efficacy of different probiotics in premature infants, finding that the combined administration of *Lactobacillus*, *Bifidobacterium*, and prebiotics may help to decrease the mortality and morbidity rates associated with NEC and sepsis [211]. Regardless of the potential benefits, specific strains’ safety and effectiveness must be thoroughly evaluated before administration to preterm neonates, due to their vulnerable health status [212].

The American Academy of Pediatrics does not support the regular administration of probiotics in premature infants, citing a lack of conclusive evidence and the absence of FDA regulation, underlining the need for further research to optimize probiotic use in this population [213]. For preterm infants, the Committee on Nutrition of the ESPGHAN and its Working Group on Probiotics and Prebiotics advise the utilization of *L. rhamnosus GG* (LGG) ATCC 53103 (with daily doses between 1 × 10^9^ CFU to 6 × 10^9^ CFU) and a combination of *B. infantis* Bb-02, *B. lactis* Bb-12, and *S. thermophilus* TH-4 (daily dose of 3.0 to 3.5 × 10^8^ CFU for each strain), as these may lower the incidence of NEC stage 2 or 3 in preterm newborns (though with low certainty in evidence). However, there’s no definitive conclusion on their impact on mortality and sepsis [113,212].

The creation of minimal or synthetic microbiotas, mirroring those found in human milk, is proposed as an innovative strategy to shield the premature infant group from NEC and sepsis, thereby enhancing the survival prospects of preterm babies at earlier stages of gestation [214].

To summarize, probiotics, prebiotics, and symbiotics represent promising interventions to reduce morbidity and mortality among preterm infants [207,210,215,216]. Despite encouraging results, the optimal strains, dosages, and routes of administration remain under investigation. Ongoing research will be crucial in consolidating the role of these therapies, developing precise guidelines for their use, and ultimately improving health outcomes for preterm infants.

## 7. Conclusions

The infant gut microbiome, a diverse ecosystem of microorganisms, is fundamental to health and well-being throughout life. This complex microbial community is established early in life and undergoes significant changes during infancy. The composition of the infant gut microbiome is influenced by several factors, mostly by diet and other nutrition strategies.

There is increasing evidence that the maternal diet during pregnancy may influence the initial seeding of the infant gut microbiome. While supplementation with probiotics and prebiotics during pregnancy may have some initial effects, it does not appear to have a long-lasting impact on the infant’s gut microbiota. This suggests that a balanced, nutritionally adequate maternal diet may be more important for healthy infant gut microbiome development rather than supplementation alone. Feeding methods in infancy are critical in shaping the gut microbiota. Breastfeeding promotes the growth of *Bifidobacteria*, contributing to the health of the infant, while formula-fed infants exhibit a more diverse but less stable microbiome, often featuring a higher prevalence of *Clostridiales* and *Proteobacteria*. Advances in formula composition, such as the addition of HMOs and prebiotics, are closing this gap, with some formulas resulting in gut microbiota that more closely resemble those of breastfed infants.

When it comes to the introduction of complementary foods, the timing and diversity of foods are of key importance. The transition from a *Bifidobacterium*-dominant community to one dominated by *Bacteroidetes* and *Firmicutes* aligns with this dietary shift. Hence, introducing a diverse range of solid foods can contribute to a well-balanced gut microbiota.

Preterm infants appear to be a specific niche in this context. Born earlier than 32 weeks of gestation, they exhibit lower microbial diversity and a higher abundance of *Proteobacteria*. However, when fed their mother’s breast milk, the gut microbiota of preterm infants becomes more diverse and abundant in beneficial bacteria such as *Bifidobacteria* and *Lactobacilli*. This suggests that breast milk plays a critical role in fostering a healthier gut microbiota, even in preterm infants.

Existing research in this field has several limitations. One significant constraint is the heterogeneity of the studies, which includes variations in the methods used for microbiota determination, dietary recording and the countries involved. Additionally, studies often lack longitudinal designs that would allow for understanding the long-term impacts of early microbiome development. There is also a need for more diverse and large-scale population studies to better understand the variations in gut microbiota across different ethnicities, geographies, and lifestyles. Furthermore, the mechanistic links between the gut microbiome and specific health outcomes in infants are still not fully understood. This diversity in study designs and methodologies presents a challenge in synthesizing a comprehensive understanding of the subject, thus representing another limitation to our current knowledge base. Taken together, these findings and future research directions underscore the need for nutritional guidance during pregnancy, the promotion of breastfeeding where possible, careful consideration of formula composition, and the thoughtful introduction of solid foods. For preterm infants, encouraging maternal breast milk feeding could significantly improve gut microbiota development. Further research should focus on these areas to enhance our understanding and develop effective interventions.
